# Mr. Collins, on Tinct. Ferri Muriati

**Published:** 1806-09

**Authors:** John Charles Collins

**Affiliations:** Swansea


### *To the Editors of the Medical and Physical Journal.*

GENTLEMEN,

ON July 12, I was sent for and consulted by a patient, attended by Dr. H. and Mr. L. of this town, labouring under excruciating pain from a retention of urine from stricture. I found him extremely irritable, pulse 110, weak, tongue dry, bowels regular; and also that he had taken freely of the ext. cicutæ and opium, as well as having been in the hot bath; that the catheter could not be introduced, nor a bougie; and from the history of the case, the patient, a captain of a vessel, living an irregular life, aged about sixty, and afflicted with this complaint for above fifteen years; that he had taken much medicine, particularly antispasmodics. The nature of the case did not allow of much delay, nor did he seem likely to be relieved but by an operation, which I was unwilling to perform, as neither of the above gentleman could be found. I therefore, at the earnest entreaties of the patient that I would do something for his relief, recollected a case Mr. Cline mentions in his Lectures, wherein the tinct. ferri muriati produced unexpected relief. I immediately procured some, which I began giving gt. v. every six minutes, in a little wine and water. After the third and fourth dose, the patient became quieter, and, in less than half an hour, some water passed without any pain. In one hour he had discharged above half a pint, and continued to do so for some time with great relief; the other medical gentlemen then arriving, a strong dose of opium was given, and he spent a tolerable night.

I did

I did not see him again for two days, when, upon being sent for, I found him reduced to the same deplorable state as before, although he had taken very large quantities of the cicuta and opium. We then repeated the tincture, and in less than two hours the man was again relieved by a discharge of urine. The first time he took about ninety drops, altogether; the last time about one hundred and thirty. I am, &c.

*Swansea, August* 14, 1806. JOHN CHARLES COLLINS.

